# Insect succession patterns on pig carrion in southern Nigeria

**DOI:** 10.1007/s12024-025-00990-0

**Published:** 2025-04-02

**Authors:** Izuchukwu Stanley Etoniru, Desiré Brits, Jolandie Myburgh, Maryna Steyn, Lawrence Hill

**Affiliations:** 1 Human Variation and Identification Research Unit, Department of Anatomical Sciences, School of Biomedical Sciences, Faculty of Health Sciences, University of the Witwatersrand, Johannesburg, South Africa; 2 Department of Anatomy, School of Medicine, Faculty of Health Sciences, University of Pretoria, Pretoria, South Africa; 3 Department of Forensic Medicine, School of Clinical Medicine, Faculty of Health Sciences, University of the Witwatersrand, Johannesburg, South Africa

**Keywords:** Insect succession patterns, Forensic entomology, Post-mortem interval, Decomposition, Southern Nigeria

## Abstract

In analyzing decomposing human remains in cases of unattended death, observing insect succession patterns to aid in estimating the post-mortem interval (PMI) based on carrion insects is one of the tasks of the forensic entomologist. The purpose of this study is to provide baseline data in order to improve PMI estimates using carrion insects. The lingering armed conflict in Nigeria creates a situation where unidentified human remains overwhelm law enforcement agencies. A common challenge is the lack of affordable, easy-to-use, and locally derived methods. This study aims to describe the succession patterns of arthropods, including insects, in pig carcasses in the wet and dry seasons in southern Nigeria as a baseline to aid in PMI estimation. Arthropods were observed and collected (all life cycle stages) in a total of 20 freshly-killed pigs (10 pigs for each season) which were deployed to study sites within 3 h of humane killing and at various times during the two seasons over 14 months. The time of appearance, activity, and disappearance of the arthropods were noted and related to the stages of decomposition to create succession patterns. Three classes, six orders, and 16 families of arthropods were collected. *Musca domestica* was the first to arrive, followed by *Chrysomya marginalis*, visiting within 10 min after placement. An ant species (Family Formicidae) had the widest presence through decomposition stages. There was an increase in the absolute number and species richness of arthropods in the wet season. Some arthropods, like the black soldier fly (*Hermetia illucens*), were exclusively present in the wet season. The observed succession patterns can be used as a reference for forensic scientists to aid in PMI assessment in Nigeria. Arthropods found exclusively in a season could be used to establish the season of death.

## Introduction

The identification of human remains is of great concern in Nigeria due to an increase in violent crimes and armed conflict in the past decade. A dearth of forensic experts in Nigeria; and the consequent absence of affordable methods of investigation adapted to the local setting leads to a situation where law enforcement agents perform mass burials without victim identification [1]. This creates an extensive list of missing persons who may have been buried without the knowledge of their families. The magnitude of this problem was confirmed in a 2022 report by the International Committee of the Red Cross (ICRC), stating that 25,000 (39%) of the 64,000 reported missing persons across Africa are Nigerians and about 56% (14,000) of the missing persons in Nigeria are children [2].

The initial step in victim identification is assessing the time between the death and discovery of the decedent, or the post-mortem interval (PMI). When the PMI is accurate, it allows for investigation into the cause and manner of death, verification of witness testimony and reduces the number of possible victims [3]. Therefore, a need exists to find affordable and easy-to-use methods for PMI estimation in a resource-limited country like Nigeria.

Insects are ubiquitous and the earliest visitors to decomposing remains. Their community composition and succession patterns allow for PMI estimation due to the definite pattern of appearance and disappearance of the various insects that visit the carcass as they interact with the changing food source and with one another [4]. This succession pattern coincides with the stages of decomposition [5–7]. This is valuable when the PMI is suspected to run into months or years [8, 9]. Since insect community composition varies between ecological environments, studies on succession patterns from different countries and regions are important to establish local standards for PMI estimations [10–22].

Unfortunately, research in forensic entomology is scarce in Nigeria [23, 24], particularly in the southeastern part of the country. There is thus a need for local entomological data that may be applied to PMI estimation. Although some studies have been done [12, 20, 24–27], they covered a short portion of a season [20, 24, 27], used other animals that are not comparable to humans in terms of decomposition [20, 25, 26], or were mostly conducted in other regions of the country [26, 27]. This is the first large scale study in southern Nigeria that considers arthropod succession patterns in pig carcasses at different times during the wet and dry seasons, including the periods of transition between seasons. The aim of this study was thus to delineate the insect succession patterns as related to the various phases of decomposition in both the wet and dry seasons in southern Nigeria. This provides baseline data pertaining to the insect succession patterns in carrion in southern Nigeria and gives guidelines on the indigenous entomofauna of forensic importance as well as their time of appearance and duration of activity for the estimation of PMI in southern Nigeria. This data will enable an affordable method for PMI estimation in a resource-limited environment.

## Materials and methods

**Fig. 1:**
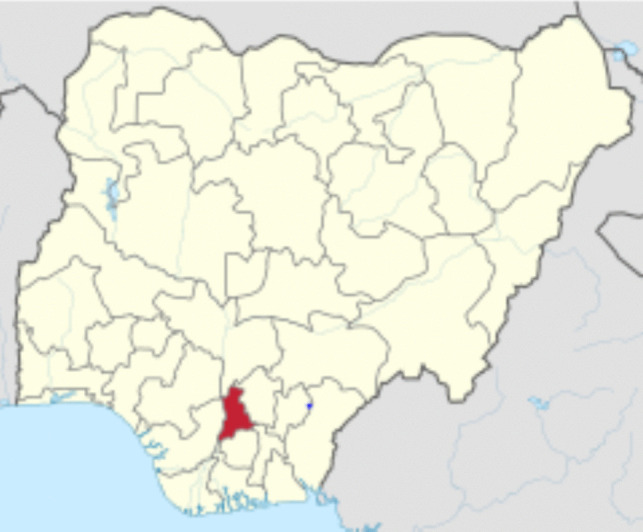
The location of Anambra State where the research site is located (Map of Nigeria, 28 February 2023 from https://en.wikipedia.org/wiki/Anambra_State)

This study was conducted at a fallow private property in Nibo (6°10′0″ N, 7°4′0″ E), a small town in Anambra state, in the south of Nigeria (Fig. 1). This property is at an altitude of 123 m (403.54 ft) above sea level.

The tropical climate of Nigeria has wet and dry seasons, as opposed to winter and summer seasons in many other parts of the world. Commonly the wet season lasts longer (April to October) than the dry season (November to March). Most of southern Nigeria has a hot, humid climate due to persistently high temperatures and its proximity to water bodies, unlike the mostly hot and arid north that is closer to the Sahara Desert. Southern Nigeria has temperature and relative humidity ranges of 20 °C—37 °C (68°F—98.6 °F) and 70 – 100%, respectively [28]. Nibo has an average temperature of 26.9 °C (80.4°F), average wind speed of 7.5 km/h (4.7 mph), and minimum and maximum humidity that ranges between 35 and 90%. The average rainfall is 1862 mm per year. The change in daily mean temperatures throughout the year is about 3.6 °C (38.1°F). The average temperature and rainfall in the wet season are 26.3 °C (79.3°F) and 199 mm, while in the dry season it is 27.5 °C (81.5°F) and 37.8 mm, respectively (World Weather Online and Climate-Data).

Ethical clearance for this research was obtained from the Animal Research Ethics Committee, University of the Witwatersrand (2019/08/46/A) and the Research and Ethics Committee (REC) of the Veterinary Services Department of the Ministry of Agriculture and Rural Development in Nigeria (MOA/ANV/441/Vol. 1/40).

Twenty freshly killed domestic pigs (*Sus scrofa*) of the large white breed were used for this study. The pigs were killed by butchers with a knife-stab wound to the heart and were purchased after inspection to ensure that there were no other external wounds. To minimize the effect of body size on decomposition rate, the weights were kept between 30 and 66 kg [29, 30]. The slaughter wound on the chest area was reconstituted with nylon suture size 2 (Agary Pharmaceuticals), cleaned, and sealed with a fabric medical plaster. This was done to reduce the effect of a more rapid insect colonization on the wound and a resultant overall expedited decomposition [31–34]. The pigs were put in plastic bags for transportation (maximum duration of ± 1 h) and taken to the study site for deposition within three hours after death.

Each pig was numbered and placed in a chicken mesh metal cage (120 cm x 80 cm x 60 cm) in direct sun. The cages were anchored to the ground to protect them from scavenger disturbance and allowed the pig carcasses to be in contact with the ground due to the open base.

A minimum distance of 10 m was maintained between cages to prevent arthropod cross-colonization [35]. Ten pigs were deposited at different times during the dry and wet seasons respectively (*n* = 20). The pigs were deployed in different seasons to observe the differences in insect activity occasioned by season. Once skeletonization was reached, the remains were removed from the cage, the cage was moved to a new site and a new pig carcass was deposited.

During a pilot study it was observed that decomposition progressed very rapidly with insect activity peaking in the first four to six days. Data collection thus proceeded as follows: daily during the first week after deploying the pig, once every three days in the second week, then every second week until the late stages of advanced decomposition or skeletonization were reached. During each visit, insects were collected and the Total Body Score (TBS) of each pig was recorded, in order to be able to associate the stage of decomposition with the insects observed. The TBS is the sum of the points (based on the five stages of decomposition) assigned to the three body regions, viz. head and neck, trunk and limbs, by visual assessment of the extent of decomposition [2]. The TBS scoring was done by using the amendments for pigs to Megyesi et al*.’*s method [3] by Keough et al*.* [36].

Adult flies were collected by aerial sweeps using a hand net. As decomposition progressed, larvae and beetles were collected using forceps. Caution was exercised to collect only two or three of a particular arthropod for identification in order not to alter the course or duration of the decomposition process. The collected insects and beetles were killed using an ethyl acetate killing jar and pinned for identification [37]. The larvae were divided into two groups: one group was reared to adult stage on ground chicken liver in an improvised insect cage for identification, while the other was immersed in hot water for five minutes and fixed in 70% ethyl alcohol for preservation and subsequent identification. Photographs of the collected arthropods and larvae were sent to, and identified by, the senior author LH, an entomologist at the Department of Forensic Medicine of the University of the Witwatersrand as no such specialist could be found in Nigeria. Insects of forensic importance were identified to the genus and species level whereas incidental insects were identified to the lowest morphological taxonomic level.

The arthropods collected were grouped into classes, orders and families to determine the number of these groups that were represented in the study. Furthermore, the insect succession patterns obtained from this identification were compared between the wet and the dry seasons. It was also compared to other studies on the continent, mostly from South Africa as this is where the majority of comparable studies on the continent were conducted. This study was mostly descriptive (qualitative) in nature.

## Results

**Fig. 2:**
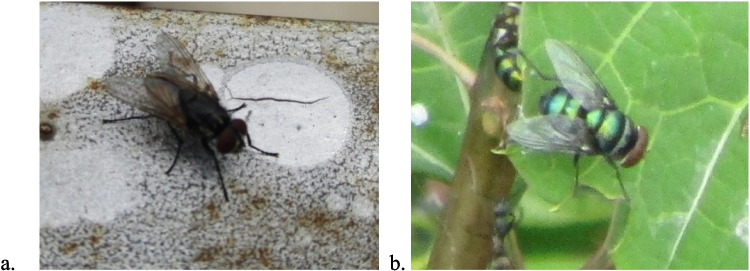
The various flies present during the fresh decomposition stage; **a** *Musca domestica*, Family Muscidae and **b** *Chrysomya marginalis*, Family Calliphoridae

The fresh stage of decomposition during both the wet and dry seasons lasted for only one day. The necrophagous housefly*, Musca domestica* (De Geer, 1776), was mostly the first to arrive during this stage followed closely by the blow fly *Chrysomya marginalis* (Wiedemann, 1830). These arthropods visited within 5 min and 10 min after placement, respectively (Fig. 2).

**Fig. 3:**
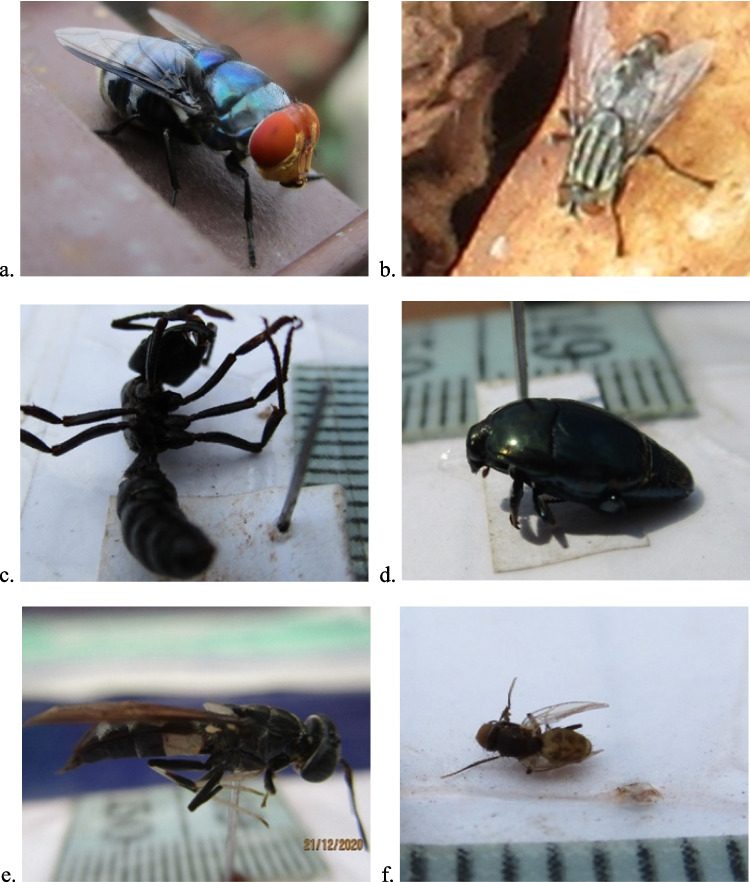
The various insects present during the bloat stage including **a** *Chrysomya chloropyga*, Family Calliphoridae, **b** Flesh fly, Family Sarcophagidae, **c** ant, Family Formicidae, **d** beetle, Family Gyrinidae, **e** Black soldier fly/*Hermetia illucens*, Family Stratiomyidae, and **f** the fly, Family Phoridae

The bloat stage lasted between days two and three after placement (TBS ranging from five to 14). The necrophagous insects associated with this stage were limited and included *Chrysomya chloropyga* (Wiedemann, 1818), observed on the second and third days, and *Sarcophaga sp.* (Meigen, 1826) (Family: Sacrophagidae) appearing from day three (Fig. 3a and b, respectively). Other predacious and omnivorous insects that visited the pig carcassess during the bloat stage included an ant species (Family Formicidae) on day two, beetles of the Family Gyrinidae, the black soldier fly (*Hermetia illucens* (Linnaeus, 1758)) and flies of the necrophagous Family Phoridae. These all appeared on day three (Fig. 3c to f). The beetles of the family Gyrinidae and the black soldier flies were exclusively present in the wet season and were observed until day 11. The flies of the Phoridae family were found in both seasons and were present until about day 22. Overall, Calliphoridae was the most dominant necrophagous family, reaching a peak appearance by the third to fourth day, or the time of maximum bloat. They gradually thinned out and eventually disappeared between the fourth and seventh days.

The beginning of the active decay stage was generally marked by newly hatched maggots from eggs deposited in body orifices during the fresh stage. As desiccation set in and progressed, the developed larvae began to move away from the pig carcasses to pupate due to the changing food resource. This migration coincided with the end of the active decay stage. We found the active decay stage to last between days four and six after placement (TBS of 20 to 24). The maggots were observed to favour the area immediately surrounding the carcass, in order to burrow into the soil and pupate in the dry season. They moved further away from the remains in the wet season. Consequently, far more new flies and pupal casings were observed within or around the cage during the dry season than in the wet season.

**Fig. 4:**
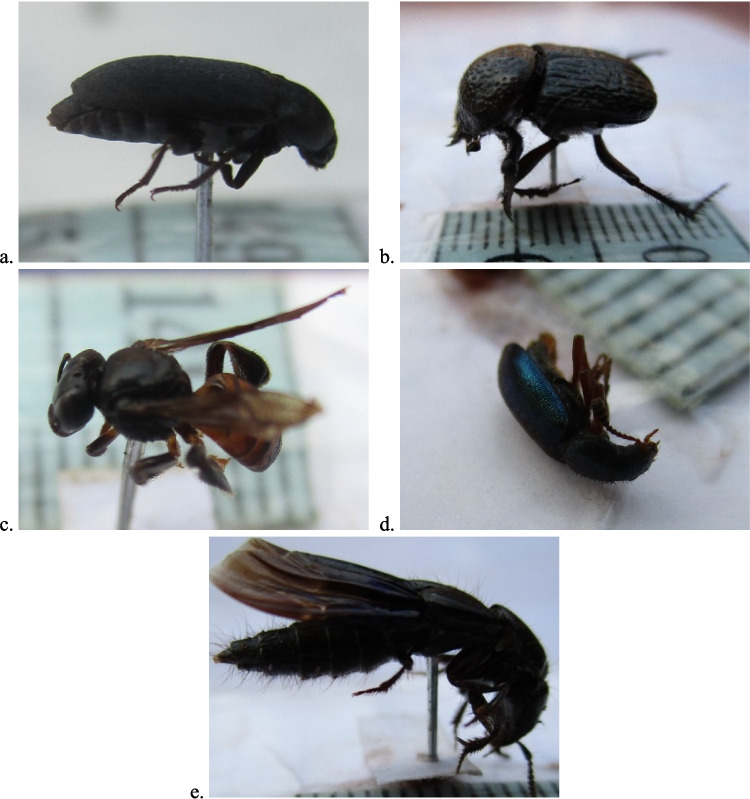
The various insects present during the active decay stage: **a** *Dermestes maculatus* beetle, Family Dermestidae, **b** scarab beetle, Family Scarabaeidae, **c** bee, Family Apidae, **d** *Platycorynus dejeani* beetle, Family Chrysomelidae and **e** rove beetle, Family Staphylinidae

The active decay stage arthropods were mainly beetles. Necrophagous Dermestid beetles, *Dermestes maculatus* (DeGeer, 1774) (Fig. 4a), appeared between the fourth and the sixth day and lasted for a variable time depending on the season; up to the 16th day in the dry season and 30th day in the wet season. Adventive Scarab beetles (Family Scarabaeidae) and bees (Family Apidae) appeared only briefly between the fourth and fifth days (Fig. 4 b and c, respectively). However, whereas the scarab beetles were exclusively observed in the wet season, bees were common in both seasons. Another adventive beetle species, *Platycorynus dejeani* (Chevrolat, 1836) (Family Chrysomelidae), appeared on day four while the predacious rove beetle (Family Staphylinidae) was observed in only one wet season pig between days six and 16 (Fig. 4 d and e, respectively).

**Fig. 5:**
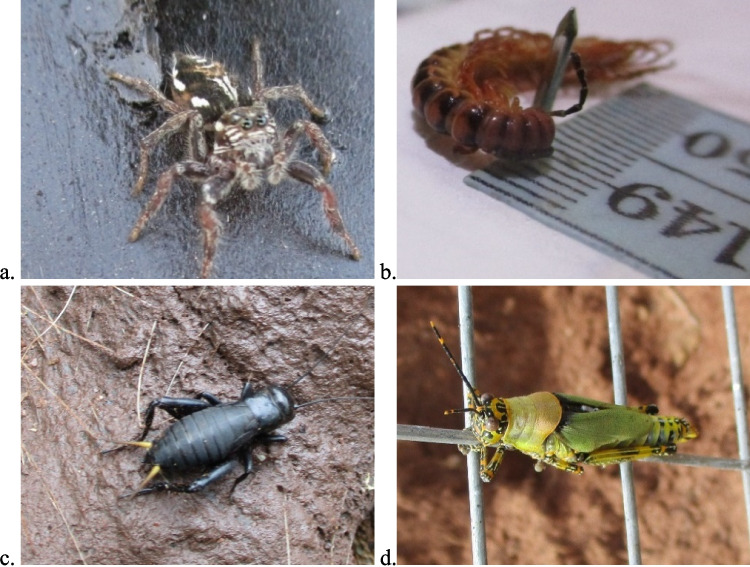
The various incidental insects present during advanced decay and dry stages of decomposition: **a** Jumping spider, Family Salticidae; **b** centipede, Family Scolopendridae; **c** cricket (*Gryllus sp.* (Linnaeus, 1758)), Family Grylloidea; and **d** variegated grasshopper (*Zonocerus variegatus* (Linnaeus, 1758)), Family Pyrgomorphidae

All the stages of decomposition described above (i.e., the earlier stages) were found not to differ in duration between the seasons. Although starting around day seven after placement, the advanced decay stage was found to be considerably shorter in the wet season than the dry season. Starting around day 7 for both seasons, it ended between days 51 and 107 in the wet season (Table 1) with a TBS range of 20 to 35, while it was observed to end between days 118 and 190 after placement in the dry season (Table 1) with a TBS range of 18 to 33. Materializing later in the decomposition process, were predacious jumping spiders (Family Salticidae), predacious centipedes, adventive crickets and grasshoppers (Fig. 5), appearing after two weeks of placement. These arthropods used the carcasses as shelter. These arthropods, along with the bees which appeared early, are incidentals and are therefore not forensically important. However, very few of the forensically important arthropods, such as *Musca domestica* and the flesh flies, which were observed during the earlier stages, were also found in these late stages (Figs. 2a and 3b).

**Table 1:** Summary of the adult arthropods associated with the stages of decomposition in the wet and dry seasons (‘ + ’ denotes present; ‘- ‘ denotes absent

Wet season	Stages of decomposition				
Family	Fresh (Day 0–1) (TBS 0–3)	Bloat (Day 2–3) (TBS 4–14)	Active (Day 4–6) (TBS 12–25)	Advanced (Day 7–107) (TBS 20–31)	Dry (> Day 51) (TBS ≥ 24)
Muscidae	+	+	+	+	-
Calliphoridae	+	+	+	-	-
Formicidae	-	+	+	+	+
Chrysomelidae	-	-	+	+	+
Gyrinidae	-	+	+	+	-
Stratiomyidae	-	+	+	+	-
Phoridae	-	+	+	+	-
Dermestidae	-	-	+	+	-
Scarabaeidae	-	+	+	-	-
Staphylinidae	-	-	+	+	-
Apidae	-	+	+	-	-
Salticidae	-	-	-	+	-
Dry season	Stages of decomposition				
Family	Fresh (Day 0–1) (TBS 0–3)	Bloat (Day 2–3) (TBS 5–11)	Active (Day 4–6) (TBS 12–22)	Advanced (Day 7–190) (TBS 18–33)	Dry (> Day 118) (TBS ≥ 24)
Muscidae	+	+	+	+	+
Calliphoridae	+	+	+	+	-
Formicidae	-	+	+	+	-
Chrysomelidae	-	-	+	+	-
Phoridae	-	+	+	+	-
Dermestidae	-	-	+	+	-
Apidae	-	+	+	-	-

For the group of pig carcasses placed during the first dry season (December 21, 2019 – March 16, 2020), there was a noticeable interlude in insect activity which coincided with late advanced decomposition. During this time the remains were completely desiccated. However, with the rehydration of the remains in the wet season, there was a return of some insects to continue decomposition which eventually led to skeletonization. These insects were much fewer in number than during early decomposition and included necrophagous *M. domestica*, Sarcophagids and a few calliphorids. There were also some larvae and a few larvae predators such as the ants.

Ants (Family Formicidae) and beetles, especially *P. dejeani* (Family Chrysomelidae), were found to scavenge on the maggots and the newly emerged flies which emerged around day 13. The time of appearance of these arthropods and their duration of stay on the carcasses coincided with the presence of these insect food sources. Both families (Formicidae and Chrysomelidae) typically stayed on or visited the carcass until either complete desiccation in the dry season, or rapid skeletonization in the wet season which made the food resource unsuitable for maggots. It is important to point out the role of arthropods in facilitating skeletonization by removing chunks of desiccated tissues hydrated during the wet season.

The dry season witnessed fewer families of arthropods than the wet season—there were seven families during the dry season and twelve during the wet season (Table 1). Apart from this obvious difference in the number of arthropod families present for each season, there was an observed increase in the absolute number of insects noted in the wet season when compared to those in the dry season. The arthropods collected from the decomposing pig carcasses included three classes, six orders, and 16 families (Table 2). The arthropods’ duration on the carcasses was longer in the wet season.

**Table 2:** Arthropods observed during pig decomposition according to classes, orders, and families

Class	Order	Family	Stage of decomposition: Fresh—F, Bloat—B, Active—Ac, Advanced—Ad, Dry—D	
			Wet season	Dry season
Insecta	Diptera	Muscidae *Musca domestica* Calliphoridae *Chrysomya marginalis* *Chrysomya chloropyga* Stratiomyidae *Hermetia illucens* Phoridae Sarcophagidae	F, B, Ac, Ad F, B, Ac B B, Ac, Ad B, Ac, Ad B	F, B, Ac, Ad, D F, B, Ac, Ad B - - B
	Coleoptera Hymenoptera Hemiptera Orthoptera	Chrysomelidae *Platycorynus dejeani* Gyrinidae Dermestidae *Dermestes maculatus* Scarabaeidae Staphylinidae Formicidae Apidae *Meliponula sp* Grylloidea Acrididae Pyrgomorphidae	Ac, Ad, D B, Ac, Ad Ac, Ad B, Ac Ac, Ad B, Ac, Ad, D B, Ac Incidental Incidental Incidental	Ac, Ad - Ac, Ad - - B, Ac, Ad B, Ac Incidental Incidental Incidental
Arachnida	Araneae	Salticidae	Incidental	Incidental
Chilopoda			Incidental	Incidental

## Discussion

Insects are the first animals to arrive at decomposing remains, and exert their effect by spreading bacteria, and larval feeding which accounts for the greatest amount of soft tissue loss during decomposition [32, 38, 39]. Due to differences in species and behaviours of these arthropods among regions and between seasons, and paucity of data in Nigeria, it was important to observe, collect and identify these arthropods to produce a comprehensive data set of insects of forensic importance. Their succession patterns over the two seasons of the year were also observed. In this study, the succession patterns were compared between the wet and dry seasons, and with other studies.

### Diptera succession

During the fresh stage of decomposition, *Musca domestica* was often the first fly to arrive at the scene in both seasons followed closely by *Chrysomya marginalis*. This contradicts observations in other studies in southern Nigeria and elsewhere, in which Calliphorids arrived before Muscids [22, 27, 40]. Ekanem and Dike [27], following an earlier study using domestic pigs at the end of the dry season in south eastern Nigeria, reported late arrival of Muscidae in the bloat stage. This difference could be due to microenvironmental or habitat differences [41] as the location of the earlier study was near a ravine which saw different fauna that included crustaceans. A study in the north-central part of Nigeria has shown that a marked difference exists in succession patterns between dry land and regions proximal to water bodies [42]. A previous study in south-eastern Nigeria [27] was carried out between March and April, a period which could be described as a transition period between the dry and the wet seasons and therefore may not represent typical conditions in either the dry or wet seasons. Decomposition has been demonstrated to be more rapid during this transition period by Etoniru et al., (submitted). Also, the short period over which succession was captured in this earlier study [27] may not reflect the entire succession dynamics. The arrival of the first flies within minutes of deposition in the current study could be exploited for more precise timing of succession intervals. A study in Jos, the central region of Nigeria, which utilised rabbit carcasses, found other species of the Calliphoridae like *Lucilia cuprina, Chrysomya albiceps, Chrysomya megacephala* and *Phormia regina* to be the first colonizers, closely followed by the common housefly *Musca domestica* in the fresh stage [42]. Carcass size and species difference, as well as differences in environment and seasons, may play a part in the observed differences in insect activity with the present study. Jos has a lower average daily temperature of 23 – 25 ◦C even in the dry season when the study was performed [42]. Another study in the Kirimiro region of Burundi [43] which utilized mouse carcasses, observed *Monomorium pharaonis* and *Leptothorax acervorum*, both of the Family Formicidae, to be the first insects to visit the carcasses and by far the most dominant. This could perhaps be explained by differences in carcass species and size [44–46].

Like the present study, Kelly et al*.* [47] found that the first arthropod to visit the remains in Bloemfontein, central South Africa, during winter were the Muscids (*Musca domestica*) followed by Calliphorids. Unlike the present study, however, which recorded both types of flies in the fresh stage, Kelly et al. [47] found the Calliphorids appearing for the first time in the bloated stage. This could be because the carcasses in the Kelly et al. study were wrapped in medium-weight cotton sheeting. Another study in KwaZulu-Natal, South Africa [48], found that Calliphorids like *Chrysomya marginalis*, *Chrysomya chloropyga,* and *Chrysomya putoria* were the first visitors and that they appeared before the Muscids (*Musca domestica*). However, they all appeared within a few hours during the fresh stage. Similar to previous studies, the bloat stage was associated with the highest number of Dipterans [48–50]. This has been reported to be due to the strong odour and their preference for soft tissue degradation caused by both autolysis and putrefaction [51, 52]. In the current study, the period of activity of the *Chrysomya* species up to the advanced stage during the dry season are similar to the finding in KwaZulu-Natal [48].

The blow fly was the most significant forensic fly in both the present study and that in Ghana [22], although it was *Chrysomya marginalis* in Nigeria and *Chrysomya rufificacies* in Ghana. The difference could be due to factors such as competition, food quality, the prevailing insect species and predation which are important factors that affect carrion insect availability and succession [41, 53–55]. The finding of more insect families and species in the dry season of Ghana [22] underlines the positive influence of humidity on insect abundance and decomposition rate as humidity was unusually marginally higher during the dry season when compared with the wet season.

The newly emerged flies from reared larva collected during this study in the active decomposition stage were identified as *Lucilia sericata*, the common blow fly. Interestingly, the adult stage of this insect was not observed at the research site throughout the period of study unlike other members of Calliphoridae like *Chrysomya marginalis* and the rare *Chrysomya chloropyga*. This may be due to the nocturnal activity and oviposition of *Lucilia sericata* which was reported by an earlier study in the United States [56] and in north-western India [57]. Considering that arthropod sampling for this study was performed during the day, the adult fly may not have been sampled. This needs further research as earlier studies indicate that *Lucilia sericata* is widely distributed in Nigeria [20, 58, 59].

### Coleopteran succession

Chrysomelidae (*Platycorynus dejeani*) were the most dominant beetles in the present study, appearing in active decomposition and staying until skeletonization was reached during the wet season, or advanced decomposition during the dry season. This was followed by Dermestidae (*Dermestes maculatus*) which were present between the active decomposition and early advanced decomposition of both seasons. *Platycorynus dejeani* did not appear in South African studies [47, 48, 60], but the presence of *Dermestes maculatus* in the active stage agrees with the findings of Kelly et al. [47] in central South Africa but not in KwaZulu-Natal [48] where they appeared earlier in the bloat stage. Unlike the present study in which the activity of *Dermestes maculatus* ended in advanced decomposition, it persisted until the dry remains stage in both KwaZulu-Natal and central South Africa [47, 48]. This could be due to the prolonged advanced decomposition with desiccation during which time the carcasses usually consisted of bones covered with desiccated hide which may be too tough for the beetles. Studies in South Africa [47, 48, 60] agree that *Dermestes maculatus* is a dominant beetle species in carrion decomposition. Scarabaeidae appeared in the bloat stage in the present study and in KwaZulu-Natal [48].

### Insect activity and season

The arthropod succession patterns of the wet and dry seasons were similar. However, there were obvious differences in the absolute number and species composition which were both increased in the wet season. Those exclusively present in the wet season included the families Gyrinidae, Stratiomyidae (*Hermetia illucens*) and Scarabaeidae (Table 1). This difference in arthropod population composition and absolute number according to season or time of the year is documented in the literature [41, 48, 54, 61, 62] and has been attributed to climatic factors such as temperature and humidity [48, 53, 61–63]. This seasonal difference is important as the insects found on the remains may indicate the season of death which also affects decomposition rate [9, 18, 64].

Arthropod larvae also acted for longer during the wet season as the remains remained moist for a longer period than during the dry season. This ensured that insects like the Formicids which scavenged on larva, also acted longer in the wet season.

For the pig remains placed during the first dry season, there was a break in insect activity in late advanced decomposition at which time the tissues of the remains were completely desiccated. With the rehydration of the remains during the wet season, there was return of some insects, including those of forensic importance like *Musca domestica* and *Chrysomya marginalis*. This insect activity was of a much smaller scale. This resumption of decomposition following rehydration of initially desiccated remains was also found in Pretoria, South Africa [65].

### Rainfall and insect activity

With respect to the effect of rainfall on insect activity, both the present study and the study in Pretoria, South Africa, [65] found that active rainfall episodes reduced insect numbers. However, the insects returned to continue their activity when the remains were no longer waterlogged. Overall, there was more insect activity during the wet season of Nigeria and the summer in the Highveld (Pretoria) of South Africa. The summer season of the Highveld region is, therefore, similar to the wet season in Nigeria in that there was rainfall, increased humidity, and increased insect activity.

The species of insects that are common to both Nigeria and South Africa appear to be those that are found in the warmer summers in South Africa [41, 66, 67]. This is probably because Nigeria has no winter-like season which reiterates the effect of various climatic factors in the species composition and abundance of carrion arthropods.

One of the limitations of this study was the inability to identify all the insects to the species level due to significant challenges in finding, and willingness of, local entomologists. The insects were thus identified by an entomologist at the Department of Forensic Medicine of the University of the Witwatersrand (LH), using photographs of the insects. Hopefully, this research will stimulate interest in the potential contributions of insects in forensic contexts in Nigeria.

## Conclusion

This study provides the first data on long term decomposition and associated insect succession patterns in southern Nigeria including various times through the wet and dry seasons. The common housefly *Musca domestica* was the first to visit the remains, within 5 min after deposition, followed about five minutes later by *Chrysomya marginalis*, a blow fly, in both seasons. This short period of colonization could be exploited for more precise timing of succession intervals when these flies are found in remains. Some arthropods, like beetles of the Family Gyrinidae and the black soldier fly (*Hermetia illucens*), visited the pig carcasses exclusively in the wet season. When these insects or their pupal casings are recovered from remains, it could indicate that early decomposition occurred in the wet season. Finally, a list of the arthropods of forensic importance and the arthropod succession patterns in southern Nigeria was described which could serve as a reference and a starting point for forensic entomological research in Nigeria. Considering the regularity of the appearance and disappearance of these arthropods, their period of activity and the predilection of some of them for a specific decomposition stage or season, a fairly accurate estimation of the PMI can be attempted by comparing these variables with similar entomological data obtained from recovered remains in the same locality. The current poor application between research outcomes and application to casework can be improved through collaboration between researchers and the criminal justice system who are directly responsible for death investigations.

## Key points

1. The fresh, bloat and active decay stages of decomposition lasted for the same duration for both seasons: fresh – one day, bloat – two days, active decay – three days.
2. The necrophagous species *Musca domestica* and *Chrysomya marginalis* were the first to arrive (within 10 min) in both seasons. This early arrival could be exploited for more precise timing of succession intervals when these flies are found in remains.
3. In addition to a general increase in the total number and species richness of arthropods in the wet season, some arthropods were observed exclusively in the wet season. Their presence in remains could be used to establish the season of death due to the indication that early decomposition occurred in the wet season.
4. There was a noticeable interlude in insect activity in the dry season during which time the remains were completely desiccated. This coincided with late advanced decomposition [Total Body Score (TBS) ≥ 24].
5. The entomological data could be utilized as a reference for forensic scientists to aid in attempting a PMI assessment in Nigeria.

## Data Availability

Data can be made available upon request, following the necessary institutional permisisons and agreements.
